# (*S*)-3-Chloro-4-(4-ethyl­piperazin-1-yl)-5-[(1*R*,2*S*,5*R*)-2-isopropyl-5-methyl­cyclo­hex­yloxy]furan-2(5*H*)-one

**DOI:** 10.1107/S1600536810026929

**Published:** 2010-07-14

**Authors:** Jian-Hua Fu, Zhao-Yang Wang, Kai Yang, Chao-Xu Mao

**Affiliations:** a School of Chemistry and Environment, South China Normal University, Guangzhou 510006, People’s Republic of China

## Abstract

The title compound, C_20_H_33_ClN_2_O_3_, was obtained *via* a tandem asymmetric Michael addition–elimination reaction of 3,4-dichloro-5-(*S*)-(l-menth­yloxy)furan-2(5*H*)-one and 1-ethyl­piperazine in the presence of potassium fluoride. The mol­ecular structure contains an approximately planar five-membered furan­one ring [maximum atomic deviation = 0.024 (2) Å] and two six-membered rings adopting chair conformations. Weak inter­molecular C—H⋯O hydrogen bonding is present in the crystal structure.

## Related literature

The title compound is a 4-amino-2(5*H*)-furan­one derivative. For the biological activity of 4-amino-2(5*H*)-furan­ones, see: Kimura *et al.* (2000 ▶); Tanoury *et al.* (2008 ▶). For the asymmetric Michael addition reactions of 2(5*H*)-furan­ones, see: Bertrand *et al.* (2000 ▶); He *et al.* (2006 ▶); Sarma *et al.* (2007 ▶). For the synthesis of the title compound, see: Song *et al.* (2009 ▶).
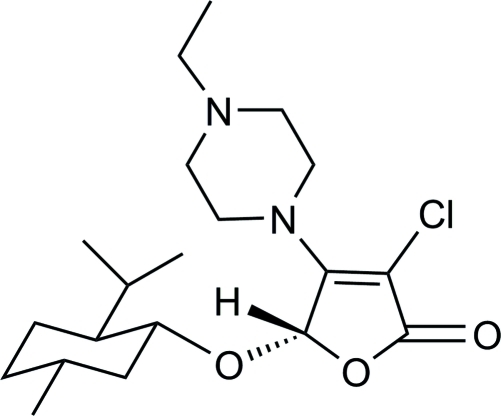


## Experimental

### 

#### Crystal data

C_20_H_33_ClN_2_O_3_
*M* *_r_* = 384.93
Orthorhombic, 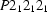

*a* = 8.7168 (15) Å
*b* = 10.1470 (18) Å
*c* = 24.478 (4) Å
*V* = 2165.1 (6) Å^3^
*Z* = 4
Mo *K*α radiation
μ = 0.20 mm^−1^
*T* = 298 K
0.23 × 0.20 × 0.16 mm

#### Data collection

Bruker APEXII area-detector diffractometer
Absorption correction: multi-scan (*SADABS*; Sheldrick, 1996 ▶) *T*_min_ = 0.956, *T*_max_ = 0.969
12202 measured reflections
4384 independent reflections
2730 reflections with *I* > 2σ(*I*)
*R*_int_ = 0.045

#### Refinement

*R*[*F* ^2^ > 2σ(*F* ^2^)] = 0.046
*wR*(*F* ^2^) = 0.107
*S* = 1.01
4384 reflections
240 parameters
H-atom parameters constrained
Δρ_max_ = 0.13 e Å^−3^
Δρ_min_ = −0.17 e Å^−3^
Absolute structure: Flack (1983 ▶), 1866 Friedel pairs
Flack parameter: 0.00 (8)

### 

Data collection: *APEX2* (Bruker, 2008 ▶); cell refinement: *SAINT* (Bruker, 2008 ▶); data reduction: *SAINT*; program(s) used to solve structure: *SHELXS97* (Sheldrick, 2008 ▶); program(s) used to refine structure: *SHELXL97* (Sheldrick, 2008 ▶); molecular graphics: *ORTEP-3 for Windows* (Farrugia, 1997 ▶); software used to prepare material for publication: *SHELXL97*.

## Supplementary Material

Crystal structure: contains datablocks global, I. DOI: 10.1107/S1600536810026929/xu2787sup1.cif

Structure factors: contains datablocks I. DOI: 10.1107/S1600536810026929/xu2787Isup2.hkl

Additional supplementary materials:  crystallographic information; 3D view; checkCIF report

## Figures and Tables

**Table 1:** Hydrogen-bond geometry (Å, °)

*D*—H⋯*A*	*D*—H	H⋯*A*	*D*⋯*A*	*D*—H⋯*A*
C4—H4⋯O2^i^	0.98	2.53	3.361 (4)	142

Symmetry code: (i) 
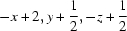
.

**Fig. 1.**
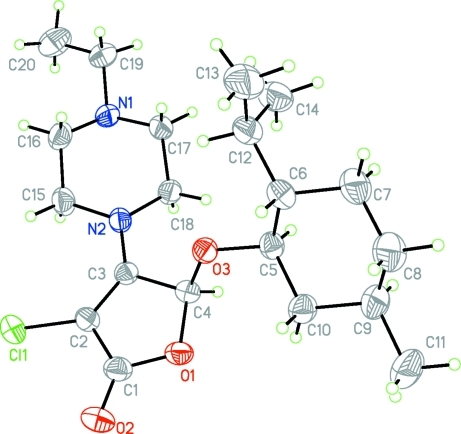
The molecular structure of the title compound showing the atom-labelling scheme. Ellipsoids are drawn at the 50% probability level.

**Fig. 2.**
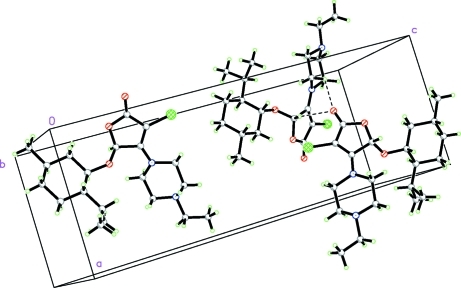
Perspective view of the crystal packing.

## supplementary crystallographic information

### Comment

With their poly-functional groups and highly active reactivity, 5-menthyloxy-2(5H)-furanones, serving as a kind of important building blocks, were widely used for the synthesis of a variety of chiral 5-menthyloxy-2(5H)-furanone derivatives. Until now, the asymmetric Michael addition reactions of 2(5H)-furanone with nucleophiles, to construct C-X (X=N, O, S, P, C) bond, have been a prominent objective in furanone chemistry (Bertrand et al., 2000; He et al., 2006; Sarma et al., 2007). At the same time, 4-amino-2(5H)-furanone is an attractive moiety in chemical, pharmaceutical and agrochemical research (Kimura et al., 2000; Tanoury et al., 2008).

Therefore we are interested in the tandem Michael addition-elimination reaction of the chiral synthon 3,4-dichloro-5-(S)-(l-menthyloxy)-2(5H)-furanone and 1-ethylpiperazine in the present of potassium fluoride. The structure of the title compound (I) is illustrated in Fig. 1. The crystal structure of the title compound which has four chiral centers ( C4(S), C5(R), C6(S), C9(R)) contains a five-membered furanone ring and two six-membered rings connected each other via C4—O3—C5 ether bond and C3—N2 bond. The furanone ring of C2—C3—C4—O1—C1 is approximately planar, whereas the six-membered ring displays a chair conformation.

### Experimental

The precursor 3,4-dichloro-5-(S)-(l-menthyloxy)-2(5H)-furanone was prepared according to the literature procedure (Song et al., 2009). After the mixture of 3,4-dichloro-5-(S)-(l-menthyloxy)-2(5H)-furanone (2.0 mmol) and potassium fluoride (6.0 mmol) was dissolved in absolute tetrahydrofuran(2.0 mL) under nitrogen atmosphere, tetrahydrofuran solution of 1-ethylpiperazine (3.0 mmol) was added. The reaction was carried out under the stirring at room temperature for 24 h. Once the reaction was complete, the solvents were removed under reduced pressure. The residual solid was dissolved in dichloromethane. Then the combined organic layers from extraction were concentrated under reduced pressure, and the crude product was purified by silica gel column chromatography with the gradient mixture of petroleum ether and ethyl acetate to give the product yielding (I) 0.280 g (36.1%).

### Refinement

H atoms were positioned in calculated positions with C—H = 0.93-0.98 Å and were refined using a riding model, with U_iso_(H) = 1.5U_eq_(C) for methyl and 1.2U_eq_(C) for the others.

### Crystal data

**Table:**

C_20_H_33_ClN_2_O_3_	*F*(000) = 832.0
*M**_r_* = 384.93	*D*_x_ = 1.181 Mg m^−^^3^
Orthorhombic, *P*2_1_2_1_2_1_	Mo *K*α radiation, λ = 0.71073 Å
Hall symbol: P 2ac 2ab	Cell parameters from 1893 reflections
*a* = 8.7168 (15) Å	θ = 2.6–19.0°
*b* = 10.1470 (18) Å	µ = 0.20 mm^−^^1^
*c* = 24.478 (4) Å	*T* = 298 K
*V* = 2165.1 (6) Å^3^	Block, colourless
*Z* = 4	0.23 × 0.20 × 0.16 mm

### Data collection

**Table:**

Bruker APEXII area-detector diffractometer	4384 independent reflections
Radiation source: fine-focus sealed tube	2730 reflections with *I* > 2σ(*I*)
graphite	*R*_int_ = 0.045
φ and ω scan	θ_max_ = 26.3°, θ_min_ = 1.7°
Absorption correction: multi-scan (*SADABS*; Sheldrick, 1996)	*h* = −10→10
*T*_min_ = 0.956, *T*_max_ = 0.969	*k* = −8→12
12202 measured reflections	*l* = −30→30

### Refinement

**Table:**

Refinement on *F*^2^	Secondary atom site location: difference Fourier map
Least-squares matrix: full	Hydrogen site location: inferred from neighbouring sites
*R*[*F*^2^ > 2σ(*F*^2^)] = 0.046	H-atom parameters constrained
*wR*(*F*^2^) = 0.107	*w* = 1/[σ^2^(*F*_o_^2^) + (0.0425*P*)^2^ + 0.0387*P*] where *P* = (*F*_o_^2^ + 2*F*_c_^2^)/3
*S* = 1.01	(Δ/σ)_max_ < 0.001
4384 reflections	Δρ_max_ = 0.13 e Å^−^^3^
240 parameters	Δρ_min_ = −0.17 e Å^−^^3^
0 restraints	Absolute structure: Flack (1983), 1866 Friedel pairs
Primary atom site location: structure-invariant direct methods	Flack parameter: 0.00 (8)

### Special details

**Table:**

Experimental. Data for (I): [α]^20°^_D_ = -32.5° (c 0.452, CH_3_CH_2_OH); ^1^H NMR (400 MHz, CDCl_3_, TMS): 0.720 (3H, *d*, *J* = 6.8 Hz, CH_3_), 0.766-1.142 (12H, *m*, CH, CH_2_, 3CH_3_), 1.221-1.388 (2H, *m*, 2CH), 1.611-1.660 (2H, *m*, CH_2_), 2.103-2.227 (2H, *m*, CH_2_), 2.435 (2H, *d*, *J* = 7.2 Hz, CH_2_), 2.495-2.515 (4H, *m*, 2CH_2_), 3.484-3.548 (1H, *ddd*, *J* = 4.4 Hz, *J* = 4.4 Hz,*J* = 4.4 Hz,CH), 3.591-3.624 (2H, *m*, CH_2_), 3.729-3.761 (2H, *m*, CH_2_), 5.751 (1H, *s*, CH), ESI-MS, *m*/*z* (%): Calcd for C_20_H_34_ClN_2_O_3_^+^([M+H]^+^): 385.22, Found: 385.39 (72.0).
Geometry. All esds (except the esd in the dihedral angle between two l.s. planes) are estimated using the full covariance matrix. The cell esds are taken into account individually in the estimation of esds in distances, angles and torsion angles; correlations between esds in cell parameters are only used when they are defined by crystal symmetry. An approximate (isotropic) treatment of cell esds is used for estimating esds involving l.s. planes.
Refinement. Refinement of F^2^ against ALL reflections. The weighted R-factor wR and goodness of fit S are based on F^2^, conventional R-factors R are based on F, with F set to zero for negative F^2^. The threshold expression of F^2^ > 2sigma(F^2^) is used only for calculating R-factors(gt) etc. and is not relevant to the choice of reflections for refinement. R-factors based on F^2^ are statistically about twice as large as those based on F, and R- factors based on ALL data will be even larger.

### Fractional atomic coordinates and isotropic or equivalent isotropic displacement parameters (Å^2^)

**Table:**

	*x*	*y*	*z*	*U*_iso_*/*U*_eq_	
Cl1	0.92355 (9)	−0.19339 (9)	0.15783 (3)	0.0824 (3)	
C1	1.0114 (3)	−0.1277 (3)	0.25997 (11)	0.0600 (7)	
C2	0.8953 (3)	−0.1150 (3)	0.21895 (10)	0.0527 (7)	
C3	0.7814 (3)	−0.0351 (2)	0.23643 (9)	0.0464 (6)	
C4	0.8249 (3)	0.0073 (3)	0.29384 (9)	0.0486 (6)	
H4	0.8300	0.1035	0.2967	0.058*	
C5	0.7051 (3)	0.0215 (3)	0.38183 (9)	0.0526 (6)	
H5	0.6699	0.1120	0.3756	0.063*	
C6	0.5819 (3)	−0.0524 (3)	0.41368 (10)	0.0604 (7)	
H6	0.6217	−0.1414	0.4200	0.072*	
C7	0.5633 (4)	0.0115 (4)	0.46984 (11)	0.0893 (10)	
H7A	0.5220	0.0997	0.4653	0.107*	
H7B	0.4903	−0.0391	0.4911	0.107*	
C8	0.7140 (4)	0.0195 (4)	0.50074 (12)	0.1017 (12)	
H8A	0.6975	0.0648	0.5351	0.122*	
H8B	0.7494	−0.0690	0.5089	0.122*	
C9	0.8364 (4)	0.0913 (4)	0.46850 (12)	0.0842 (10)	
H9	0.8015	0.1820	0.4626	0.101*	
C11	0.9897 (4)	0.0966 (4)	0.49839 (12)	0.1230 (15)	
H11A	0.9775	0.1439	0.5321	0.185*	
H11B	1.0640	0.1407	0.4760	0.185*	
H11C	1.0241	0.0086	0.5060	0.185*	
C12	0.4297 (3)	−0.0685 (3)	0.38336 (12)	0.0701 (8)	
H12	0.4543	−0.1031	0.3470	0.084*	
C13	0.3255 (4)	−0.1697 (4)	0.41080 (14)	0.1061 (13)	
H13A	0.3810	−0.2504	0.4161	0.159*	
H13B	0.2378	−0.1859	0.3880	0.159*	
H13C	0.2921	−0.1365	0.4455	0.159*	
C14	0.3423 (4)	0.0598 (4)	0.37461 (14)	0.0934 (11)	
H14A	0.2544	0.0436	0.3519	0.140*	
H14B	0.4081	0.1229	0.3571	0.140*	
H14C	0.3092	0.0938	0.4093	0.140*	
N2	0.6474 (2)	0.0035 (2)	0.21506 (8)	0.0520 (5)	
C15	0.5990 (3)	−0.0392 (3)	0.16045 (10)	0.0684 (8)	
H15A	0.6371	−0.1275	0.1535	0.082*	
H15B	0.6425	0.0192	0.1332	0.082*	
C16	0.4266 (3)	−0.0384 (3)	0.15570 (10)	0.0656 (8)	
H16A	0.3968	−0.0654	0.1192	0.079*	
H16B	0.3831	−0.1005	0.1815	0.079*	
N1	0.3681 (2)	0.0924 (2)	0.16674 (9)	0.0557 (6)	
C17	0.4034 (3)	0.1223 (3)	0.22317 (10)	0.0628 (8)	
H17A	0.3579	0.0559	0.2466	0.075*	
H17B	0.3591	0.2069	0.2328	0.075*	
C18	0.5741 (3)	0.1263 (2)	0.23249 (11)	0.0581 (7)	
H18A	0.6178	0.1995	0.2123	0.070*	
H18B	0.5944	0.1406	0.2710	0.070*	
C19	0.2032 (3)	0.1040 (3)	0.15658 (12)	0.0750 (9)	
H19A	0.1686	0.1891	0.1698	0.090*	
H19B	0.1503	0.0367	0.1774	0.090*	
C20	0.1589 (4)	0.0909 (4)	0.09789 (13)	0.0967 (12)	
H20A	0.2230	0.1468	0.0760	0.145*	
H20B	0.0536	0.1165	0.0934	0.145*	
H20C	0.1715	0.0010	0.0865	0.145*	
O3	0.71812 (18)	−0.04391 (16)	0.32993 (6)	0.0511 (4)	
O1	0.97260 (19)	−0.05115 (19)	0.30335 (7)	0.0606 (5)	
O2	1.1286 (2)	−0.1903 (2)	0.25964 (9)	0.0842 (7)	
C10	0.8549 (3)	0.0261 (3)	0.41285 (10)	0.0655 (8)	
H10A	0.9300	0.0747	0.3916	0.079*	
H10B	0.8931	−0.0629	0.4178	0.079*	

### Atomic displacement parameters (Å^2^)

**Table:**

	*U*^11^	*U*^22^	*U*^33^	*U*^12^	*U*^13^	*U*^23^
Cl1	0.0705 (5)	0.0943 (6)	0.0825 (5)	0.0125 (5)	0.0059 (4)	−0.0335 (5)
C1	0.0460 (16)	0.0667 (19)	0.0674 (18)	−0.0018 (15)	0.0059 (15)	−0.0001 (15)
C2	0.0478 (16)	0.0528 (17)	0.0576 (16)	0.0012 (14)	0.0048 (13)	−0.0056 (13)
C3	0.0434 (14)	0.0465 (16)	0.0492 (14)	−0.0033 (13)	0.0018 (12)	0.0005 (12)
C4	0.0420 (14)	0.0505 (16)	0.0532 (15)	0.0018 (12)	0.0031 (11)	0.0020 (13)
C5	0.0592 (16)	0.0538 (16)	0.0448 (14)	0.0042 (15)	0.0001 (12)	−0.0004 (13)
C6	0.0641 (18)	0.0607 (17)	0.0564 (16)	0.0063 (16)	0.0095 (14)	0.0025 (14)
C7	0.092 (2)	0.120 (3)	0.0563 (18)	0.002 (2)	0.0142 (17)	−0.0032 (19)
C8	0.109 (3)	0.148 (4)	0.0476 (17)	0.007 (3)	0.0026 (19)	0.005 (2)
C9	0.091 (2)	0.104 (3)	0.0579 (19)	0.005 (2)	−0.0121 (17)	−0.0102 (19)
C11	0.114 (3)	0.186 (4)	0.069 (2)	−0.008 (3)	−0.029 (2)	−0.019 (3)
C12	0.0611 (19)	0.076 (2)	0.0736 (18)	−0.0031 (18)	0.0139 (16)	−0.0076 (17)
C13	0.092 (3)	0.111 (3)	0.115 (3)	−0.026 (2)	0.037 (2)	−0.002 (2)
C14	0.069 (2)	0.111 (3)	0.101 (3)	0.019 (2)	0.0029 (18)	−0.004 (2)
N2	0.0535 (13)	0.0548 (13)	0.0477 (12)	0.0074 (11)	−0.0039 (9)	−0.0076 (10)
C15	0.074 (2)	0.079 (2)	0.0519 (16)	0.0173 (17)	−0.0076 (14)	−0.0158 (15)
C16	0.0697 (19)	0.069 (2)	0.0585 (16)	0.0001 (17)	−0.0149 (15)	−0.0098 (15)
N1	0.0487 (13)	0.0603 (15)	0.0582 (13)	0.0017 (11)	−0.0077 (10)	−0.0035 (12)
C17	0.0540 (17)	0.070 (2)	0.0643 (18)	0.0115 (15)	−0.0043 (14)	−0.0135 (15)
C18	0.0570 (16)	0.0541 (17)	0.0631 (16)	0.0097 (14)	−0.0130 (14)	−0.0084 (14)
C19	0.0573 (18)	0.090 (2)	0.077 (2)	−0.0004 (17)	−0.0157 (16)	−0.0069 (18)
C20	0.083 (2)	0.113 (3)	0.094 (2)	0.000 (2)	−0.0368 (19)	0.008 (2)
O3	0.0510 (10)	0.0557 (11)	0.0465 (9)	−0.0049 (9)	0.0040 (8)	−0.0008 (8)
O1	0.0435 (10)	0.0781 (13)	0.0601 (11)	0.0085 (10)	−0.0047 (8)	−0.0019 (10)
O2	0.0541 (12)	0.0954 (16)	0.1032 (17)	0.0258 (12)	−0.0054 (11)	−0.0103 (14)
C10	0.0653 (18)	0.079 (2)	0.0524 (16)	−0.0018 (17)	−0.0031 (13)	0.0017 (15)

### Geometric parameters (Å, °)

**Table:**

Cl1—C2	1.712 (2)	C12—H12	0.9800
C1—O2	1.203 (3)	C13—H13A	0.9600
C1—O1	1.358 (3)	C13—H13B	0.9600
C1—C2	1.431 (4)	C13—H13C	0.9600
C2—C3	1.351 (3)	C14—H14A	0.9600
C3—N2	1.338 (3)	C14—H14B	0.9600
C3—C4	1.518 (3)	C14—H14C	0.9600
C4—O3	1.384 (3)	N2—C18	1.463 (3)
C4—O1	1.437 (3)	N2—C15	1.468 (3)
C4—H4	0.9800	C15—C16	1.507 (4)
C5—O3	1.438 (3)	C15—H15A	0.9700
C5—C10	1.511 (3)	C15—H15B	0.9700
C5—C6	1.524 (4)	C16—N1	1.447 (3)
C5—H5	0.9800	C16—H16A	0.9700
C6—C7	1.529 (4)	C16—H16B	0.9700
C6—C12	1.529 (4)	N1—C17	1.447 (3)
C6—H6	0.9800	N1—C19	1.463 (3)
C7—C8	1.518 (4)	C17—C18	1.506 (3)
C7—H7A	0.9700	C17—H17A	0.9700
C7—H7B	0.9700	C17—H17B	0.9700
C8—C9	1.514 (5)	C18—H18A	0.9700
C8—H8A	0.9700	C18—H18B	0.9700
C8—H8B	0.9700	C19—C20	1.494 (4)
C9—C10	1.523 (4)	C19—H19A	0.9700
C9—C11	1.524 (4)	C19—H19B	0.9700
C9—H9	0.9800	C20—H20A	0.9600
C11—H11A	0.9600	C20—H20B	0.9600
C11—H11B	0.9600	C20—H20C	0.9600
C11—H11C	0.9600	C10—H10A	0.9700
C12—C14	1.524 (4)	C10—H10B	0.9700
C12—C13	1.527 (4)		
			
O2—C1—O1	121.2 (3)	C12—C13—H13C	109.5
O2—C1—C2	130.0 (3)	H13A—C13—H13C	109.5
O1—C1—C2	108.7 (2)	H13B—C13—H13C	109.5
C3—C2—C1	110.6 (2)	C12—C14—H14A	109.5
C3—C2—Cl1	131.4 (2)	C12—C14—H14B	109.5
C1—C2—Cl1	118.0 (2)	H14A—C14—H14B	109.5
N2—C3—C2	133.9 (2)	C12—C14—H14C	109.5
N2—C3—C4	119.8 (2)	H14A—C14—H14C	109.5
C2—C3—C4	106.2 (2)	H14B—C14—H14C	109.5
O3—C4—O1	110.14 (19)	C3—N2—C18	121.1 (2)
O3—C4—C3	108.46 (19)	C3—N2—C15	121.4 (2)
O1—C4—C3	104.89 (19)	C18—N2—C15	113.0 (2)
O3—C4—H4	111.1	N2—C15—C16	110.8 (2)
O1—C4—H4	111.1	N2—C15—H15A	109.5
C3—C4—H4	111.1	C16—C15—H15A	109.5
O3—C5—C10	113.0 (2)	N2—C15—H15B	109.5
O3—C5—C6	106.3 (2)	C16—C15—H15B	109.5
C10—C5—C6	111.5 (2)	H15A—C15—H15B	108.1
O3—C5—H5	108.6	N1—C16—C15	110.0 (2)
C10—C5—H5	108.6	N1—C16—H16A	109.7
C6—C5—H5	108.6	C15—C16—H16A	109.7
C5—C6—C7	109.0 (2)	N1—C16—H16B	109.7
C5—C6—C12	114.6 (2)	C15—C16—H16B	109.7
C7—C6—C12	112.9 (2)	H16A—C16—H16B	108.2
C5—C6—H6	106.6	C17—N1—C16	107.2 (2)
C7—C6—H6	106.6	C17—N1—C19	110.7 (2)
C12—C6—H6	106.6	C16—N1—C19	112.9 (2)
C8—C7—C6	112.2 (3)	N1—C17—C18	111.1 (2)
C8—C7—H7A	109.2	N1—C17—H17A	109.4
C6—C7—H7A	109.2	C18—C17—H17A	109.4
C8—C7—H7B	109.2	N1—C17—H17B	109.4
C6—C7—H7B	109.2	C18—C17—H17B	109.4
H7A—C7—H7B	107.9	H17A—C17—H17B	108.0
C9—C8—C7	112.1 (3)	N2—C18—C17	111.4 (2)
C9—C8—H8A	109.2	N2—C18—H18A	109.4
C7—C8—H8A	109.2	C17—C18—H18A	109.4
C9—C8—H8B	109.2	N2—C18—H18B	109.4
C7—C8—H8B	109.2	C17—C18—H18B	109.4
H8A—C8—H8B	107.9	H18A—C18—H18B	108.0
C8—C9—C10	109.4 (3)	N1—C19—C20	114.2 (3)
C8—C9—C11	112.6 (3)	N1—C19—H19A	108.7
C10—C9—C11	110.6 (3)	C20—C19—H19A	108.7
C8—C9—H9	108.0	N1—C19—H19B	108.7
C10—C9—H9	108.0	C20—C19—H19B	108.7
C11—C9—H9	108.0	H19A—C19—H19B	107.6
C9—C11—H11A	109.5	C19—C20—H20A	109.5
C9—C11—H11B	109.5	C19—C20—H20B	109.5
H11A—C11—H11B	109.5	H20A—C20—H20B	109.5
C9—C11—H11C	109.5	C19—C20—H20C	109.5
H11A—C11—H11C	109.5	H20A—C20—H20C	109.5
H11B—C11—H11C	109.5	H20B—C20—H20C	109.5
C14—C12—C13	109.8 (3)	C4—O3—C5	116.3 (2)
C14—C12—C6	114.3 (3)	C1—O1—C4	109.41 (19)
C13—C12—C6	112.0 (3)	C5—C10—C9	111.8 (2)
C14—C12—H12	106.8	C5—C10—H10A	109.3
C13—C12—H12	106.8	C9—C10—H10A	109.3
C6—C12—H12	106.8	C5—C10—H10B	109.3
C12—C13—H13A	109.5	C9—C10—H10B	109.3
C12—C13—H13B	109.5	H10A—C10—H10B	107.9
H13A—C13—H13B	109.5		

### Hydrogen-bond geometry (Å, °)

**Table:**

*D*—H···*A*	*D*—H	H···*A*	*D*···*A*	*D*—H···*A*
C4—H4···O2^i^	0.98	2.53	3.361 (4)	142

Symmetry codes: (i) −*x*+2, *y*+1/2, −*z*+1/2.
